# Direct Anterior Tracks: Early and Functional Management of Class III Malocclusions—Case Report and Literature Review

**DOI:** 10.1155/2019/9323969

**Published:** 2019-06-27

**Authors:** Echeverry Juan Carlos, Barbosa-Liz Diana

**Affiliations:** ^1^Faculty of Dentistry, University of Antioquia, Calle 70 #52-21 Medellín, Colombia; ^2^Orthopedic Maxillary and Orthodontic Postgraduate Program, Faculty of Dentistry, Universidad de Antioquia, Calle 70 #52-21 Medellín, Colombia

## Abstract

The prevalence of class III malocclusion ranged from 0 to 26% in different populations. Many types of treatments have been described in dental literature. The results of early treatment have been positive. The purpose of this report is to describe the case of a four-year-old patient with class III malocclusion who received an innovative treatment using direct anterior tracks. This therapy efficiently obtained immediate improvement of profile and occlusal relationships.

## 1. Introduction

The development and establishment of class III malocclusions can occur from the early stages of life, and the establishment of early functional alterations can permanently affect the growth of craniofacial structures [1–3].

Dentofacial changes achievable with maxillary orthopedics can induce harmonic growth when an early approach of these maxillofacial alterations is made [4].

### 1.1. Etiopathogenesis

Multiple factors can influence the presence of a class III malocclusion and its etiology has a multifactorial origin. The influence of inheritance on the appearance of this dysplasia and its primary etiological role have been widely reported [5, 6].

Class III malocclusion can be skeletal, dental, or functional. Alterations in the size and/or position of the maxilla and mandible may favor the establishment and development of skeletal class III malocclusion. On the other hand, the premature contact between the anterior incisors at early ages or the inadequate inclination of these teeth could be etiological factors of functional and dental class III malocclusion [4]. Likewise, other functional and epigenetic factors, such as lingual anterior lower position, oral breathing, and the absence of teeth on upper arch, among others, have been identified as environmental etiological factors of class III [4].

Altered eruption can induce an unfavorable incisal guide, generating an anterior crossbite. The anterior displacement of the mandible and its condyle as a consequence of unfavorable incisal guidance can produce a pseudo-class III or class III malocclusion. Likewise, the premature loss of primary molars can cause mandibular anterior displacement due to the change in the occlusal guide of the teeth that are in malposition or to the lingualization of the maxillary incisors [3]. The prevalence of class III malocclusion varies from 0.6 to 1.2% depending on the population studied. In Colombia, there is a prevalence of 5.8% of functional and pseudo-class III alterations in children of 5-17 years. The prevalence of true or skeletal class III in the same population was 3.7% [7].

Graber et al.'s recognition of the condyle's ability to adapt to different stimuli received from the growth of the mandible in malocclusions with anterior crossbite makes the functional orthopedics of the jaws a relevant value for early management of sagittal skeletal alterations [3].

The differential diagnosis of true class III, functional class III, and class I with anterior crossbite is important and it takes into account the profile, posterior dental relationships, dental inclinations, the presence or absence of anterior mandibular deflection, and the complete evaluation of the functional aspect [8].

If possible and the lateral cephalic radiograph exists, the evaluation of maxillomandibular position and size is useful. Likewise, the anterior crossbite can be a sign of different combinations of skeletal and dental discrepancies [9].

The orthopedic-orthodontic treatment will depend on the etiology and the specific characteristics of the malocclusion. The age of the patient also influences the treatment decision. Therapeutic approaches range from maxillary advance with facial mask, progenie mechanical devices, functional orthopedic appliances like Bionator and Frankel, to functional myo-dynamic orofacial orthopedics among others.

As the transversal width of the maxilla is usually affected, the treatment must take into account this aspect, using rapid maxillary expanders [10].

The systematic review of De Toffol et al. evaluated the scientific evidence of the effectiveness of orthopedic treatment with the use of facial mask, chip cup, Frankel 3, and Bionator, among others. The results described over 75% of success of treatment with rapid maxillary expansion (RME) and facial mask therapy at a follow-up observation 5 years after the end of orthopedic therapy. Regarding other therapies, they could not find enough evidence about the success and stability of the treatment [11]. Other authors have found similar findings [5, 12]. Recently, with the advance of skeletal anchorage using miniscrews, the combined treatments using bone anchorage and mechanic appliances have showed positive results [13]. However, this kind of treatments is used to be indicated for mixed dentition patient.

### 1.2. Early Vision of the Therapeutics of Anterior Crossbites

It is important that parents have children checked early for anterior crossbite treatment. Therapy should be directed toward the elimination of the etiological and conditioning factors that can contribute to anterior crossbite [14] since it interferes with the down and forward growth and displacement of the maxilla [5]. This early correction can reduce the need for further treatments (maxillary orthopedics, orthodontics, and/or maxillofacial surgery) and can allow for more optimal psychological and social development [15]. In anterior crossbites, the occlusal plane tends toward convergence with respect to the Camper plane, causing a more open angle between these planes and upsetting the balance of one of the laws of Planas in craniofacial growth.

### 1.3. Therapeutic Objectives in Early Treatment of Anterior Crossbite

The objectives of the treatment of anterior crossbites are (1) to achieve a normal overjet and generate changes in the neuromuscular activity of the chewing muscles while at rest and in function, (2) to influence the position of the condyle in the glenoid cavity, (3) to restore the posture and position of the craniocervical-hyoid complex, (4) to produce changes in the control of the morphogenetic and tissue rotation of the maxilla [16] and the mandible [17], (5) to control the position and pressure of the tongue in the orofacial apparatus, (6) to allow homeostasis in the maxillomandibular growth when there are discrepancies between them, (7) to make the centric occlusion coincide with the centric relation, thereby eliminating the occlusal or functional deflection, and (8) to attempt to parallelize the occlusal plane to the Camper plane [18]. To achieve this, we must try to achieve ideal function by changing the position of the mandible and the pattern of mandibular movements and by improving masticatory muscle activity and mandibular dynamics [19]. The clinician can explore therapeutic variables to reestablish a harmonic occlusion, such as selective cutting and/or the use of direct tracks.

Direct tracks, designed and developed by Dr. Pedro Planas [20], are a good therapeutic option in deciduous dentition and have been used in the molar sector to therapeutically restore the occlusal plane [21]. They are made with resins or acrylic of resins or acrylic of lesser and greater consistencies and strengths. Therapeutically, it is designed to generate a physiological occlusal plane with movements of laterality with freedom that allows neural excitation, rehabilitating the maxillofacial growth vectors and the function of the temporomandibular joint in the mandibular dynamics.

Contact surfaces are created between the upper and lower arches to occlude in the usual position and allow the most appropriate mandibular sliding. They aid in the anterior or posterior reposition of the mandible (depending on the inclination of the tracks) in retroposition, laterognatism, or in mandibular protrusion, and they allow rotation of the jaw in a clockwise direction. Additionally, they prevent the establishment of morphological and positional asymmetries in the maxillofacial complex in children [22]. These tracks are preserved until the molars are replaced.

Likewise, flat tracks, according to Simoes [18], rehabilitate the masticatory function and improve the contact surfaces and rub at the posterior level, stimulating growth at the transverse level of the jaws and allowing sagittal synchronized growth between the jaws. The following clinical case shows the development and use of the direct tracks proposed by Dr. Planas and clinically adapted by Dr. Simoes for the early treatment of anterior and posterior crossbites with one modification: they are placed on the anterior teeth.

There is no scientific data regarding the use of direct tracks in the anterior deciduous teeth as part of early treatment of anterior crossbites. The clinical approach of this type of therapy can contribute to the early management of these alterations as an option in the prevention of skeletal class III malocclusions.

## 2. Case Presentation

Patient, four years old, was brought to consultation for presenting anterior crossbite that, according to the mother, began with the eruption of deciduous incisors.

**Figure 1:**
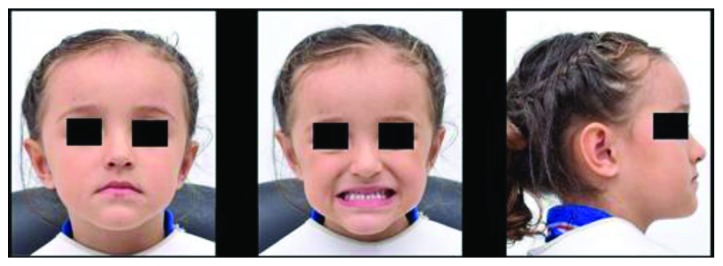
Extraoral photos.

**Figure 2:**
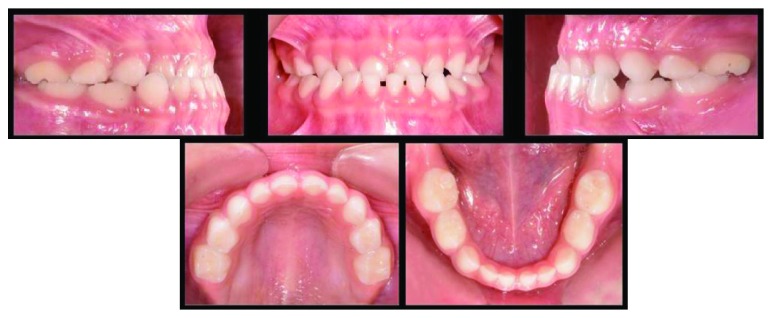
Intraoral findings.

During clinical evaluation, the patient presented a concave profile (Figure 1). Complete deciduous dentition with molar and canine class III relationships at 1 mm, an OJ of -1.5 mm, and an OB of 50% were observed (Figure 2).

**Figure 3:**
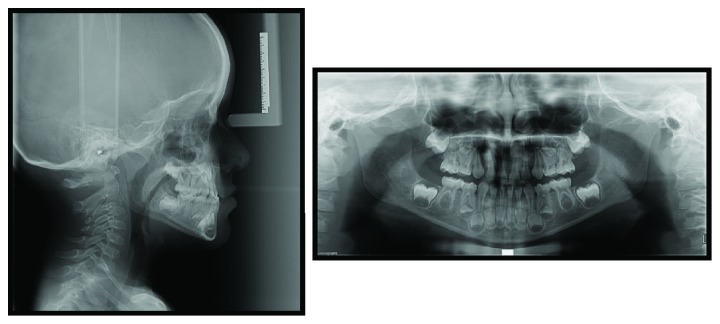
Extraoral X-rays.

The lateral cephalometric radiograph was taken in maximum intercuspidation and a hyperdivergent class III relationship with decreased ANB angle and increased mandibular body length was found. The findings of panoramic radiography were normal (Figure 3).

After a functional evaluation, the patient mandible was manipulated and carried to the front edge-to-edge relationship, and an anterior deflection with a left side deviation of the lower midline of 2 mm was observed. A diagnosis of pseudo-class III was made, as well as a decision to use cemented anterior direct tracks.

### 2.1. Impression, Bite Register, and Laboratory Procedures

**Figure 4:**
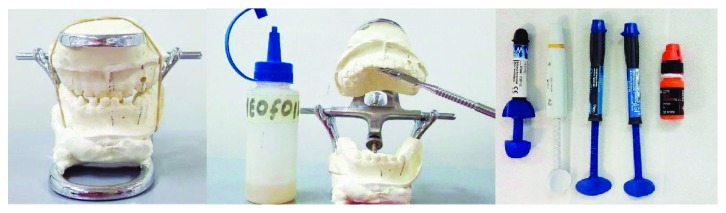
Preparing the track construction.

For the construction of the tracks, upper and lower impressions must be taken. The bite registration must be as retrusive as the mandible can be. Then, it must be mounted on a hinge articulator. Once assembled on the articulator, the buccal and palatal surface of the anterior superior teeth must be isolated with insulating material (neo foil) (Figure 4).

**Figure 5:**
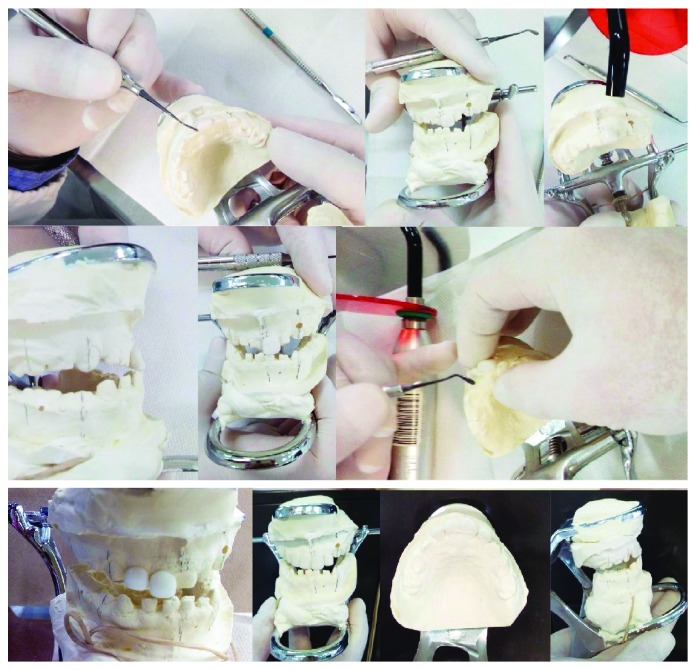
Laboratory procedures.

On the vestibular surface of the four upper incisors, individual veneers are made on each of the anterior teeth with hybrid resin with microfiller with the following specifications:

1. The incisal edge should have a thickness of 2.5 to 4 mm to prevent the closing path from being anterior. Its thickness depends on the previous slip between centric relation and centric occlusion
2. Extend 1-2 mm on the incisal in the model to create the closure guide, thickening it more towards the buccal and, at the same time, taking the incisal third towards the palatal covering with resin with a minimum possible thickness depending on the patient's closure to give a larger surface of retention to the veneer. On the laterals, make a rounded distoincisal angle to allow the patient's lateroprotrusive to be free
3. Cover the interproximal surfaces as far as the buccal surface with a razor-edge finish between the tooth surface and the resin without generating dental plaque entrapment and creating the risk of interproximal caries
4. At the gingival level, there should be a thickness of 0.5 mm, finished with a chamfer or knife edge to avoid the accumulation of dental plaque and the risk of cervical caries
5. Evaluate the closing trajectory in the articulator so that the four upper anterior teeth are left with a determinate area (DA) constructed, as much as possible, so that the patient is left without anterior crossbite (Figure 5)

### 2.2. Clinical Procedure: Cementation

**Figure 6:**
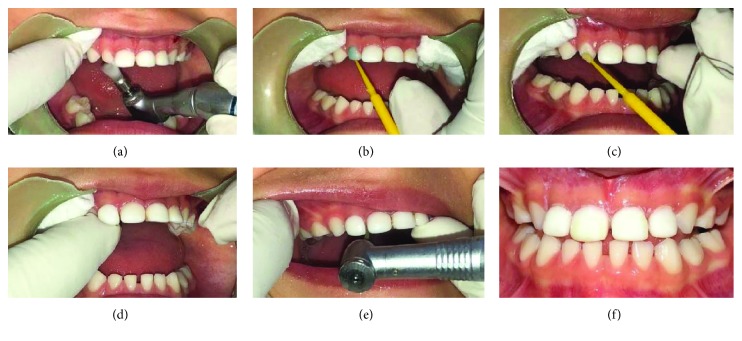
Cementation and immediate track intraoral photos.

The clinical procedure is described as follows:

1. Use prophylaxis with bicarbonate on the surfaces of the anterior teeth to remove impurities at the level of the enamel of the teeth that will serve as anchorage
2. Demineralize the surfaces of the deciduous that will serve as receptors for direct tracks with orthophosphoric acid for 30 seconds. Wash demineralized areas profusely
3. Dry the surfaces of the anterior teeth and check that the surface is white with chalk and matt tone
4. Apply bonding agent according to the manufacturer's instructions required to make the veneers on the surfaces that will serve as receptors, pass the dental floss through the interproximal surfaces, and apply the light of the curing lamp according to the manufacturer's specifications to polymerize the bonding agent
5. Apply fluid resin to each of the veneers and treat each tooth individually according to tracks designed for each tooth. Remove the excess resin from surfaces, including palatal extensions with tracks and light cures
6. Verify excess material resins at both incisal, interproximal, and cervical areas, and polish any excess resin with a finishing bur. Use metal sandpaper on the interproximal area and polish the remaining points. Keep the anatomy as similar as possible to the natural receiving teeth. Remember that the endings of the resin with the dental surfaces must be razor-sharp and have no sites that can retain dental plaque
7. Examine the lateral, protrusive, and lateroprotrusive movements. In the protrusive, the four anterior teeth should make contact to prevent fracture or the failure of the direct tracks. When doing lateral movements, there should be a disocclusion canine or group, guided by the canine and molars, that does not interfere with the anterior teeth. In lateroprotrusive movements, the distoincisal angle should be rounded to avoid interference in movement
8. An angular carving of 15 degrees or more should be made between the incisal edge towards the palatal if the occlusion allows for a posterior closure guide, so that the lower anterior teeth slide behind the upper anterior teeth when making the closure and achieve the DA necessary to provide the principles of neural excitation at the dental level (Figures 6(a)–6(f))

### 2.3. Controls

It should be made clear that during the first week the patient will have nocturnal bruxism because the vertical dimension has been increased and there is little, if any, contact in the posterior part to allow the eruption of the molars to generate a new therapeutic occlusal plane and let the jaw slide later.

Further treatment begins with periodic review appointments, the first 2-3 weeks later, and then every month. If the tracks are restored, and if necessary, the inclination of the incisal edge track towards the palatal should be prolonged and the incisal edge of the enamel can be worn to guarantee the maintenance of the DA and the mandibular position behind the maxilla.

Verify oral hygiene and reinforce oral preventive measures.

As therapeutic results and patient responses to treatment are evaluated, therapy can be reinforced with functional and orthopedic appliances.

**Figure 7:**
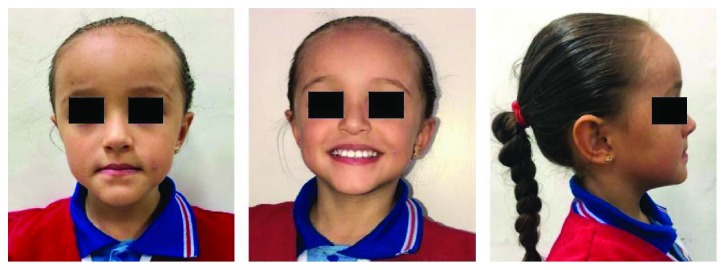
Three-month posttreatment extraoral photo.

**Figure 8:**
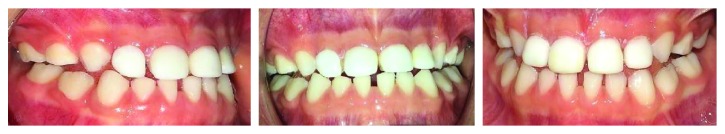
Three-month posttreatment intraoral photo.

With time, the posterior teeth will become extruded, there will be a correction in the occlusion, and the open bite will be closed. The overjet will be improved with stable and acceptable functional and aesthetic results. From the aesthetic point of view, there will be improvement in the smile line and in the exposition of incisors at rest and in sleep, and from the functional point of view, the anterior deflection will be eliminated and the appropriate occlusal function will be restored (Figures 7 and 8). The anterior direct tracks are left in position until the anterior deciduous teeth are exfoliated, controlling so that a posterior occlusion is established promoting occlusal stability. The time for the new occlusal relationships to be established was 6 months.

## 3. Discussion

Early management of class III malocclusions has been shown to have substantial influence on long-term outcome and treatment prognosis, and it has been suggested that there are short-term effects as well [23]. The sooner that results are obtained, the more efficient and stable the treatment will be [24]. This case describes the management of a class III malocclusion in a patient with deciduous dentition with the goal of creating a new occlusal plane, an adequate determinate area (DA) incisor contact, and the promotion of mandibular rotation by means of anterior direct tracks [18].

Although management with removable orthopedic appliances is effective, fixed treatment that promotes the establishment of a new mandibular position is another good therapeutic option.

Balance of the stomatognathic system structures is achieved through the aforementioned neural excitation and physiological or therapeutic mandibular posture changes [25], principles on which the techniques developed by researchers of functional appliances, such as Balters [26], Planas, Bimler, and Frankel [27], are based. These techniques act as bimaxillary by modifying the position of the jaw to obtain better and faster clinical results. Functional orthopedic devices can modify the nociceptive reflexes, favoring the development of new circuits of neuronal reflexes [18]. The results are most effective when initiating position change and when contact between deciduous incisors or between permanent teeth in the determined area (DA) are established. However, the change must be made within the physiological limits of each individual. According to Simoes, if this contact is not achieved, it makes the evolution of the chosen therapy less predictable and produces a more reserved prognosis due to the lack of neural excitement. Alterations in closure trajectory can produce instability in the condyle in the joint and deficient mandibular dynamics. But when the incisive contact (DA) is established, the results will be perceived more quickly in the efferent neuronal response component. In early management, selective carvings must be made that interfere with occlusal coupling or achieve this change in therapeutic posture [18].

The traditional tracks are made of composite resins and cemented with fluid resins in the posterior deciduous teeth in cases of transverse alterations or anterior crossbites [18] and act as functional devices as part of neuro-occlusal rehabilitation, allow returning functionality to the patient, and improve their aesthetic appearance.

Based on this, the use of direct tracks in anterior teeth is a novel and efficient therapeutic option for the management of class III malocclusions.

The use of previous direct tracks allows better marginal adaptation, visibility, and patient management, as well as better oral hygiene. Likewise, the aesthetic impact is immediate, restoring confidence and facial harmony to the patient.

It is important to remark that since upper posterior collapse and posterior crossbite problems are usually presented in class III, the evaluation and management of the transverse plane are fundamental in the treatment. In this case, specifically, the upper transversal width was just adequate but the progress of this finding needs supervision.

This article presents a novel way of approaching occlusal therapy, solving class III functional malocclusion in a simple, fast, and economical way. It may be an adequate therapeutic alternative for clinicians.
